# Psychopathy: what are fearless people afraid of?

**DOI:** 10.3389/fpsyt.2025.1574813

**Published:** 2025-06-03

**Authors:** Carlos M. Coelho, Ana S. Araújo, Panrapee Suttiwan, Fernando Barbosa, Tiago Bento, Andras N. Zsido

**Affiliations:** ^1^ Institute of Psychology, University of Pécs, Pécs, Hungary; ^2^ Life Di Center, Faculty of Psychology, Chulalongkorn University, Bangkok, Thailand; ^3^ Department of Psychology, University of the Azores, Ponta Delgada, Portugal; ^4^ University Research Center in Psychology (CUIP), Faculty of Human and Social Sciences, University of Algarve, Faro, Portugal; ^5^ Center for Psychology, University of Porto, Porto, Portugal; ^6^ Department of Social and Behavioural Sciences, University of Maia, Maia, Portugal; ^7^ Laboratory of Neuropsychophysiology, Faculty of Psychology and Education Sciences, University of Porto, Porto, Portugal

**Keywords:** primary psychopathy, secondary psychopathy, fears, phobias, diagnostic

## Abstract

**Introduction:**

Historically, the distinction between primary and secondary psychopathy has focused on fear or lack thereof and limited anxiety symptoms. Individuals high in primary psychopathy traits often exhibit little or no anxiety or fear. These traits are key features, and several methods used to differentiate primary and secondary psychopathy emphasize fear and anxiety as key discriminators. However, there is limited evidence on what individuals high in psychopathy traits might specifically fear. Most previous studies have either included specific phobias within an anxiety cluster, thereby precluding the possibility of observing the number and type of phobias reported by participants with psychopathic traits, or have addressed specific phobias in general without further detailing the specific fears to which these participants were referring.

**Methods:**

This study attempts to address this evidence gap by using the Levenson Self-Report Psychopathy Scale to measure psychopathy and the Fear Survey Schedule III to measure phobic anxiety.

**Results:**

Results indicate that individuals with higher levels of secondary psychopathy report a greater number of specific fears. In contrast, those with primary psychopathy show fear or discomfort primarily related to seeing naked people.

**Discussion:**

These findings are discussed in detail.

## 1 Introduction

The distinction between primary and secondary psychopathy was first proposed in the 1940s (1). The former, also referred to as idiopathic or constitutional psychopathy, was historically considered the “true,” “pure,” or essential type of psychopathy, also equated with a low anxiety psychopathic type (2), and later referred to as primary psychopathy. In contrast, secondary psychopathy, sometimes referred to as neurotic, symptomatic, or reactive, was thought to result from or be secondary to other psychiatric conditions, leading to terms such as “neurotic sociopath” (3) and later termed “secondary psychopathy.

The antisocial behavior often found in secondary psychopathy is similar to that of primary psychopathy, but is considered secondary to what was previously termed *neurotic conflicts* (1, 4). Contemporary conceptualizations of psychopathy maintain a two-component framework, such as the two-factor model (5, 6), where Factor I includes traits or personality characteristics such as superficial charm, lack of remorse, callousness, and egocentricity, and Factor II includes antisocial behaviors such as criminal behavior and poor behavioral control.

It has been suggested that the absence of fear and limited anxiety symptoms are key features distinguishing primary from secondary psychopathy. Hervey Cleckley (2) specifically noted this lack of fear in patients with psychopathy and suggested it as a motivating factor behind their behavior: “*When detected in activities that would cause fear, shame, or dismay in others, this patient often displayed simple insouciance*.” (p.117). Studies conducted both before and after Cleckley have consistently found that participants high in primary psychopathy traits exhibit limited, if any, anxiety symptoms (7) and often manifest low levels of anxiety (e.g., (3, 8, 9)).

The *low-anxiety hypothesis of psychopathy* has been further supported by additional research (3, 9) demonstrating differences in the acquisition of fear and anxiety between primary and secondary psychopathy, with the former showing less arousal and avoidance to the anticipation of punishment (e.g., (3, 8)). For example, Lykken (3) found that participants with primary psychopathy reported less anxiety and showed less intense physiological reactions (measured by galvanic skin response). They also showed less avoidance of a conditioned stimulus previously paired with a painful electric shock compared to two control groups (normal and secondary). A recent triarchic conceptualization of psychopathy (10) also identifies low anxiety (often referred to as boldness) as a core feature of psychopathy and the etiological processes underlying primary psychopathy. Thus, fear and trait anxiety appear to be a distinguishing feature between primary and secondary psychopathy.

Neurophysiological research has further substantiated the relationship between low anxiety and psychopathy, which has been validated by a variety of methods. These include reduced startle reflex potentiation when exposed to unpleasant pictures (11), reduced blink potentiation in anticipation of imminent threat (12, 13), reduced skin conductance responses to aversive pictures (14), and impaired fear conditioning (15). For example, Patrick (13) observed that participants with psychopathy exhibited reduced startle potentiation in response to noxious tones (110 db, 0.5 s). Similarly, they showed inhibited startle responses to scenes depicting victims (assaults or injuries) and only modestly enhanced responses to scenes depicting threats (11). Patrick and colleagues (13) also examined blink responses to sound probes in prisoners viewing affective pictures. Participants with low and moderate scores on the Psychopathy Checklist-Revised (16) showed a typical pattern (blinking inhibited for pleasant pictures and potentiated for aversive pictures), whereas participants with elevated psychopathy scores showed inhibited blinking for both pleasant and unpleasant pictures compared to neutral pictures. Overall, individuals with high primary psychopathic traits appear to show lower than normal levels of anxiety and fear responses to aversive or threatening stimuli. It’s worth noting that while the unpleasant stimuli included mutilations, pointed guns, and snakes, the authors did not separate these fears into categories, classifying them all as equally aversive.

Several methods have been proposed for the assessment of psychopathy (17). Lykken suggested that secondary psychopathy could be measured by neuroticism and developed the Activity Preference Questionnaire (18). The APQ aims to minimize psychiatric and somatic symptoms by asking, for example, whether respondents would enjoy activities such as riding an open elevator to the top of a tall building under construction or fighting a forest fire. Respondents with primary psychopathy typically score lower on anxiety responses to such situations compared to normal controls or those with secondary psychopathy. One of the most widely used scales for measuring primary and secondary psychopathy is the Levenson Self-Report Psychopathy Scale (LSRP), which assesses both subtypes of psychopathy (19). Levenson and colleagues emphasized that, particularly in non-institutionalized samples with a mixture of primary and secondary traits, anxiety may be the variable most likely to discriminate between the two subtypes.

There is limited evidence, primarily from small studies, specifically examining anxiety in individuals with psychopathy. Coid (20) examined clinical syndromes in incarcerated dangerous offenders diagnosed with “psychopathic disorder” and found that comorbidity with antisocial personality disorder was negatively associated with phobias. However, this study combined simple, social, and agoraphobia into a single category. Similarly, Pham and Saloppé (21) observed a decrease in the prevalence of phobic disorders as psychopathy scores increased. These findings are consistent with evidence suggesting that higher levels of psychopathy correlate with lower levels of anxiety. However, they do not provide further insight into the specific fears and phobias associated with psychopathy.

In summary, the primary aim of this investigation was to explore the specific fears experienced by individuals with high psychopathy traits. Specifically, we sought to determine whether there are specific fears (e.g., birds, dogs) that are more prevalent in primary compared to secondary psychopathy. Previous studies have often lumped specific phobias into broader anxiety categories, making it difficult to identify the exact fears that are most prevalent in individuals with psychopathic traits. Other studies have examined diagnostic comorbidities among inmates, but have often overlooked psychopathy or phobia subtypes. As noted above, theories of low anxiety in psychopathy have typically been examined using fear conditioning, startle reflex modulation, and self-report measures. However, no study to date has examined the specific fears that are prevalent in primary and secondary psychopathy. Given that individuals with secondary psychopathy tend to have higher levels of anxiety (22) and emotional dysregulation (23), we hypothesize that individuals high in secondary psychopathy traits will exhibit a greater number of fears compared to individuals low in psychopathy traits and individuals high in primary psychopathy traits.

With regard to primary psychopathy, the study was designed as an exploratory investigation of the specific fears experienced by individuals according to psychopathy traits. Specific hypotheses regarding individual fear items were not established at the outset, as no preferential or predefined expectations were made. The purpose of the study was to examine whether certain fear domains might still be endorsed by individuals high in primary psychopathy, despite the well-established association between primary psychopathy and low fear. The fear questionnaire used in this study (The Fear Survey Schedule III), which consists of five factors and a total of 72 items, covers a wide range of fear-related stimuli, providing an opportunity to explore potential domain-specific sensitivities. In this way, we aim to contribute to the understanding of whether certain specific fears are more likely to be associated with primary or secondary psychopathy.

## 2 Methods

### 2.1 Participants

In the present study, convenient sampling was used, with a required minimum sample size determined by *a priori* power analysis (f=0.25, 1-β=0.95) and calculated to be 252 participants by the G*Power 3 software (24).

A total of 554 Caucasian participants were recruited (177 male, 376 female, 1 preferred not to answer), aged 18-56 years *(M* = 33.2, *SD* = 12.0). The median score on the primary subscale was 28 on the Levenson Self-Report Psychopathy Scale, while the median score on the secondary subscale was 20. Individuals scoring at or above these levels were classified as having higher levels of primary and secondary psychopathy, respectively, while those scoring below these levels were classified as having lower levels of psychopathy. A total of 280 participants scored above the median on the Primary Psychopathy subscale *(M* = 34.2, *SD* = 5.79, range: 28-57), and 274 scored below the median *(M* = 23.2, *SD* = 2.77, range: 16-27). A total of 317 participants scored above the median on the secondary psychopathy subscale *(M* = 23.5, *SD* = 3.41, range: 20-39), and 237 scored below the median *(M* = 16.5, *SD* = 2.18, range: 10-19). We chose to use the median-split procedure rather than entering their scores as a continuous variable in the analysis to facilitate analytical and communicative clarity, and because the median-split procedure is more parsimonious (25).

### 2.2 Assessment

The Portuguese version of Levenson’s Self-Report Psychopathy Scale (26) was used to measure psychopathy. The scale contains 26 items that are self-rated on a 5-point Likert-type scale, with two factors measuring primary and secondary psychopathy. Higher scores indicate a greater tendency toward psychopathy. In the absence of previously published cut-off scores for these scales, a median splitting procedure was used. The McDonald’s omega for the present sample was.817 for primary psychopathy and.760 for the secondary psychopathy scale (and.83 for the total test), indicating that the questionnaire scores were reliable.

The Fear Survey Schedule III (27), adapted to the Portuguese population (28), was used to measure phobic fear. The questionnaire consists of five factors (animals, social, blood-injection-injury, noises, other) and a total of 72 items. Participants rate the items on 5-point Likert-type scales, indicating their level of fear. Higher scores indicate greater fear. The McDonald’s omega for the present sample was.97 for Total,.88 for Animal,.91 for Social,.839 for Blood Injection Injury,.75 for Noise,.81 for Other, indicating that the survey scores were reliable.

### 2.3 Procedure

An online-based survey was conducted in Portugal from January to May 2022. An anonymous online survey was developed to ensure the confidentiality of the participants and was published through social media, mailing lists and various forums to obtain a heterogeneous sample. All data was collected online and no evidence of bot responses was found.

The research was approved by the Ethical Review Committee of the University of the Azores (31/12/2021; n°54/2021) and was conducted in accordance with the Code of Ethics of the World Medical Association (Declaration of Helsinki); informed consent was obtained from all participants in the study.

### 2.4 Statistical analysis

Four duplicate responses were identified and removed prior to statistical analysis; no outliers (+3 SD criterion) and no bot responses were found. First, we created three groups based on participants’ scores on the primary and secondary psychopathy scales. Those who scored below the median on both scales were assigned to the “low psychopathy” group (N=139). Individuals who scored above the median on the Primary scale and below the median on the Secondary scale were assigned to the High Primary group (N=98). Individuals scoring above the median on the Secondary scale and below the median on the Primary scale were assigned to the High Secondary group (N=135). Group differences in anxiety were assessed using separate analyses of variance (ANOVAs) with Welch’s correction (hence the lack of effect sizes) because the assumption of homogeneity of variances was violated. ANOVAs were followed by Games-Howell corrected pairwise comparisons. Due to the large number of ANOVAs, the Benjamini-Hochberg procedure was used to reduce the false discovery rate (29), and results that violated this assumption were excluded from the final analyses. The main statistical results are presented in tabular form. Descriptive statistics and the results of the follow-up tests can be found in **Supplementary Material 1**.

## 3 Results

**Figure 1:**
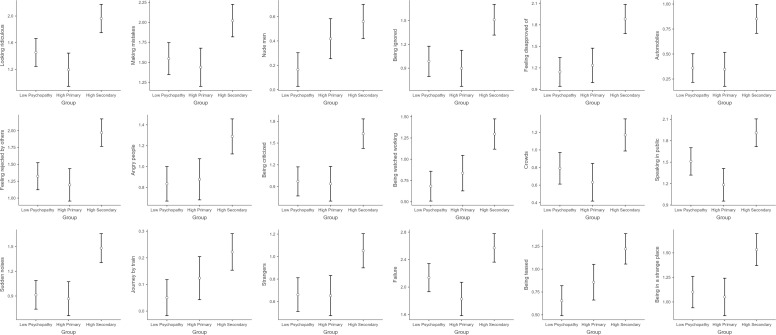
A visualization of the key findings showing the differences between the three psychopathy groups (low psychopathy, high primary, and high secondary).

A total of 18 fears survived the Benjamini-Hochberg correction, shown in **Figure 1**. Statistical results are presented in **Table 1**, for group comparisons and detailed descriptive statistics see **Supplementary Material**. In most cases, the fear scores of the Higher Secondary Psychopathy (HS) group were significantly higher than those of the Higher Primary (HP) and Lower Psychopathy (LP) groups, and the latter two did not differ from each other. These included 11 fears related to social or interpersonal situations: 1) being watched while working; 2) being criticized; 3) angry people; 4) feeling rejected by others; 5) feeling disapproved of; 6) being ignored; 7) looking ridiculous; 8) crowds; 9) speaking in public; 10) being teased; 11) strangers. One fear related to noise, namely sudden noises. And four miscellaneous fears: 1) being in a strange place; 2) making mistakes; 3) failure; 4) cars.

**Table 1:** Results of one-way ANOVAs comparing the three psychopathy groups (low psychopathy, high primary, and high secondary) on all fears on the fear survey schedule.

Fear	F	df	p	η^2^
Noise of vacuum cleaners	1.503	2, 229	0.225	0.007
Being alone	5.756	2, 234	0.004	0.033
*Being in a strange place*	*8.996*	*2, 230*	*< .001*	*0.050*
Loud voices	5.593	2, 238	0.004	0.034
*Speaking in public*	*12.297*	*2, 238*	*< .001*	*0.060*
Crossing streets	4.729	2, 228	0.010	0.029
*Automobiles*	*11.575*	*2, 229*	*< .001*	*0.073*
*Being teased*	*11.753*	*2, 225*	*< .001*	*0.059*
Dentists	3.938	2, 232	0.021	0.023
*Failure*	*9.998*	*2, 225*	*< .001*	*0.056*
Entering a room where other people are already seated	2.470	2, 227	0.087	0.014
People with deformities	1.728	2, 225	0.180	0.009
Worms	3.855	2, 234	0.023	0.021
Imaginary creatures	5.279	2, 216	0.006	0.025
Receiving injections	3.790	2, 230	0.024	0.023
*Strangers*	*7.343*	*2, 234*	*< .001*	*0.043*
Bats	4.750	2, 234	0.010	0.028
*Journey by train*	*6.891*	*2, 190*	*0.001*	*0.032*
Journey by bus	4.565	2, 221	0.011	0.027
Journey by car	5.405	2, 218	0.005	0.035
Feeling angry	1.790	2, 227	0.169	0.010
People in authority	3.231	2, 225	0.041	0.015
Flying insects	6.060	2, 233	0.003	0.034
Seeing other people being injected	3.054	2, 232	0.049	0.018
*Sudden noises*	*11.840*	*2, 230*	*< .001*	*0.070*
Dull weather	0.992	2, 234	0.372	0.006
*Crowds*	*8.181*	*2, 241*	*< .001*	*0.041*
Large open spaces	0.174	2, 229	0.840	0.001
Cats	1.529	2, 234	0.219	0.008
One person bullying another	2.269	2, 226	0.106	0.012
Tough looking people	2.491	2, 231	0.085	0.014
Birds	2.775	2, 234	0.064	0.015
Deepwater	1.178	2, 230	0.310	0.006
*Being watched working*	*11.236*	*2, 228*	*< .001*	*0.063*
Dead animals	1.270	2, 228	0.283	0.007
Weapons	1.929	2, 234	0.148	0.010
Dirt	0.830	2, 236	0.437	0.004
Crawling insects	2.475	2, 233	0.086	0.014
Sight of fighting	4.562	2, 237	0.011	0.025
Ugly people	1.135	2, 222	0.323	0.006
Fire	1.295	2, 238	0.276	0.007
Sick people	1.655	2, 228	0.193	0.009
Dogs	2.428	2, 233	0.090	0.015
*Being criticized*	*11.835*	*2, 233*	*< .001*	*0.068*
Strange shapes	5.469	2, 232	0.005	0.031
Being in an elevator	1.292	2, 237	0.277	0.008
Witnessing surgical operations	2.099	2, 237	0.125	0.012
*Angry people*	*7.744*	*2, 231*	*< .001*	*0.043*
Mice	0.484	2, 229	0.617	0.003
Human blood	0.174	2, 238	0.840	0.001
Animal blood	0.849	2, 238	0.429	0.005
Parting from friends	2.397	2, 232	0.093	0.013
Enclosed places	2.318	2, 229	0.101	0.012
*Feeling rejected by others*	*13.670*	*2, 232*	*< .001*	*0.074*
Airplanes	5.788	2, 226	0.004	0.028
Medical odors	2.847	2, 225	0.060	0.013
*Feeling disapproved of*	*13.469*	*2, 227*	*< .001*	*0.073*
Harmless snakes	2.953	2, 231	0.054	0.016
Cemeteries	2.251	2, 225	0.108	0.011
*Being ignored*	*9.534*	*2, 231*	*< .001*	*0.054*
Darkness	2.316	2, 237	0.101	0.013
Premature heartbeat/missing a beat	1.543	2, 230	0.216	0.008
*Nude men*	*8.932*	*2, 214*	*< .001*	*0.041*
Nude women	3.616	2, 208	0.029	0.017
Lightning	0.288	2, 232	0.750	0.002
Doctors	3.525	2, 227	0.031	0.020
*Making mistakes*	*7.942*	*2, 230*	*< .001*	*0.043*
*Looking ridiculous*	*11.398*	*2, 234*	*< .001*	*0.059*
Supernatural	3.208	2, 235	0.042	0.019
Bacteria/Virus	1.257	2, 232	0.287	0.007
Open wounds	2.711	2, 237	0.069	0.015
Dead people	0.908	2, 232	0.405	0.005
People who seem insane	2.089	2, 229	0.126	0.012
Falling	1.956	2, 233	0.144	0.011
Thunders	0.893	2, 233	0.411	0.005
Sirens	1.855	2, 233	0.159	0.011
High places on land	1.580	2, 235	0.208	0.009

The comparisons that survived the Benjamini-Hochberg false discovery rate correction are italicized.

In two cases, however, different results were found. For fear of traveling by train, the HP group did not differ from any of the other groups, although the LP group still scored lower than the HS group. More interestingly, for fear of naked men, both the HP and HS groups scored higher than the LP group, while the former did not differ from each other.

## 4 Discussion

There seems to be a consensus in previous studies that people with marked primary psychopathic traits have lower than normal levels of anxiety and that people with secondary psychopathy have many anxiety symptoms (2, 7–9, 19, 30). This has been shown using both self-report and physiological measures (e.g., skin response, startle reflex) (11, 13, 14). There is also evidence that primary psychopathy is negatively, whereas secondary psychopathy is positively associated with the prevalence of phobias (20, 21). However, it has remained unclear what, if anything, people with higher levels of primary and secondary psychopathy might fear most. Therefore, the present study sought to test how people with higher levels of primary and secondary psychopathy compare to a sample with low psychopathy scores in terms of various fears. Two main findings emerge from our study: first, as expected, people with secondary psychopathy report an elevated number of specific fears, particularly those related to social contexts; second, an unexpected finding, that participants with primary psychopathy report fear or discomfort at the sight of naked men. These findings will now be discussed separately.

The finding that people with higher scores on secondary psychopathy also have a high number of specific phobias (especially social phobias) is not surprising and confirms previous findings that specific phobias are strong predictors of other mood and anxiety disorders (30). In fact, multiple phobias are most commonly found in neurotic patients (31), as general neuroticism is associated with the development of multiple anxiety disorders (32). Studies of samples of offenders and people convicted of violent crimes also show that the likelihood of anxiety disorders is higher in such samples than in the general population (33). The prevalence of anxiety disorders in convicted individuals is estimated to be between 37.5% (34) and 46% (35), while the prevalence of phobias has been found to be over 35%. Regarding phobias, Bennett & Johnson (36) found positive correlations between paranoid personality disorder and specific phobia. Sareen and colleagues (37) found that the prevalence of simple phobia among participants with an antisocial diagnosis ranged from 22% to 24%.

Less expected was the finding that participants with elevated traits of primary psychopathy only seemed to be afraid of seeing naked men. Psychopaths are known to exhibit callous, dominant, and manipulative traits (38), often associated with a lack of respect for others. They lack the ability to form strong emotional bonds, experience empathy and guilt, which are associated with self-centered, irresponsible, and impulsive behavior (16, 39); they tend to use intimidation and violence to satisfy their needs (39), which are also often associated with exploitation and sexual offending (40). Correlations between sadism and psychopathy were found by Holt and Strack using clinical interviews, psychological tests, and behavioral histories (41). Socially dominant males (not females) who were able to manipulate others and had low levels of fear and anxiety also reported higher rates of risky sexual behavior (42).

Fearless, egocentric, exploitative, and impulsive individuals tend to engage in sexual behaviors with potentially harmful consequences (43). Early, coercive, and promiscuous sexual activity is common in individuals with high levels of psychopathy (44). Psychopathy is also associated with fantasies of sexual variety rather than a single partner, likely due to an inability to form attachments (45), and with a preference for short-term sexual activity outside of committed relationships (46, 47). In terms of subclinical psychopathy, participants often exhibit behaviors such as parasitism, opportunism, turbulent interpersonal relationships, and an impersonal, frivolous, and superficial sex life. These findings are consistent with other research showing that people with subclinical psychopathy have a greater number of sexual relationships outside the intimate relationship (48). van Bommel and colleagues (49) suggest that high levels of psychopathy may be associated with increased deviant sexual interests due to attachment deficits and interpersonal detachment, lack of empathy and deficits in the violence inhibition system, or an evolutionary predisposition to high mating but low parental investment (50). Taken together, these findings suggest the possibility that participants with high levels of psychopathy, although fearless overall, may have an “Achilles’ heel” when confronted with naked human intimacy.

This may be better understood using the attachment deficits and interpersonal disengagement findings associated with psychopathy. Offenders with a childhood history of physical and sexual abuse and neglect score higher on the PCL-R, as well as antisocial behavior (51). Some authors have suggested that the emotional detachment of psychopaths results from early traumatic childhood experiences of abuse, deprivation, and neglect (52). Our findings partially support studies such as that of Conradi and colleagues (53), who found that disinhibition/impulsive-irresponsible traits were positively related to attachment avoidance using the Experiences in Close Relationship Questionnaire (54), which is related to fear of rejection and abandonment. They also found that meanness/callous-unemotional traits were positively related to attachment avoidance. Similar to our study, they found unexpected results, as the grandiose/manipulative psychopathy facets were positively correlated with attachment avoidance and anxiety, suggesting an underlying experience of fear of rejection in relationships (53). Also of note is a study by Blanchard and Lyons (55) showing thatprimary psychopathic traits are related to avoidant attachment in men and to both anxious and avoidant attachment in women. In contrast, secondary psychopathic traits were predicted by anxious attachment. Similarly, Grady and colleagues (56) suggest that people who commit sexual crimes may fear intimacy in relationships, and the image of the psychopath as someone who cannot experience vulnerability and pain may be misleading (57), at least with respect to this component of intimacy, which in our study may have been elicited by asking about fear of seeing naked people.

In summary, the only fear that showed a significant positive correlation with primary psychopathy was the fear of seeing a naked man. While this may initially seem surprising given that individuals high in primary psychopathy are often described as emotionally detached and ‘fearless’, there are several possible explanations. This reaction may not reflect fear in the traditional sense, but rather discomfort in situations involving male vulnerability, ambiguous intimacy or emotional closeness between men. Such scenarios may be disturbing for individuals who value control, emotional distance and dominance — traits typically associated with the primary psychopathy profile. From an evolutionary perspective, the image of a naked man could also be perceived as a sign of physical threat or challenge, particularly in terms of dominance and competition among males. Furthermore, as previous studies have shown, some individuals with psychopathic traits, particularly those who have committed sexual crimes, may not be as indifferent to relationships as they appear. They may actually experience anxiety and emotional dysregulation in intimate contexts due to early adversity or insecure attachment (51, 56). For these individuals, discomfort in relational situations, particularly with other men, may be connected with deeper relational difficulties or past experiences of shame. Finally, it is possible that cultural and social learning, such as punishments or taboos around nudity, continues to influence people who otherwise report low levels of emotion. In this sense, although the result seems unexpected, it suggests that fear or a similar kind of discomfort can still emerge in specific situations, even in individuals who are generally considered to be emotionally cold or fearless.

Some limitations that might affect the generalisability of our study’s findings should be mentioned. Although we collected a reasonably large sample size, using a convenience sampling method may limit the strength of our conclusions. While past studies have used much smaller samples of convicted or diagnosed individuals, the results appear consistent with our own. Furthermore, in order to cover as many fears as possible, each fear was measured using a single item, which may affect the validity of the findings. Further studies collecting additional longitudinal data would provide a better grasp of this problem. Furthermore, the mechanisms involved in the novel finding of a higher fear of nude men in primary (as well as secondary) psychopathy are not entirely clear. It is also important to note that this is a correlational study and that no causal inferences can be drawn from the observed associations; all interpretations should therefore be considered exploratory. Additionally, we acknowledge that median splits have been criticised for potentially reducing statistical power and obscuring variability in continuous variables. Future research should focus on better understanding this result and exploring the same research question using continuous or data-driven classification methods.

In conclusion, our results provide new evidence that people who score higher on the secondary psychopathy scale are more anxious and fearful than those who score higher on the primary psychopathy scale (and those who score low on the psychopathy scale). Interestingly, it also seems that people scoring higher in primary psychopathy are not entirely fearless. It is yet to be decided whether this is only true for one category of fear or if it might foreshadow more subtle fears that were not studied here. Past studies have defined fearlessness as boldness and an absence of defensive responses, and have consequently studied situations where physical harm might be present. Generalising these results to all fears may be problematic, as there are numerous fears that are not directly linked to physical harm, yet which can affect individuals in more subtle ways. To fully understand the underlying mechanisms, future research should address how various stimuli that do not involve physical harm (e.g. intimacy and interpersonal distance) affect people who are prone to primary psychopathy.

## Data Availability

The datasets presented in this study can be found in online repositories. The names of the repository/repositories and accession number(s) can be found below: OSF: https://osf.io/q83fn.
