# Supplementary material for: Psychopathy: what are fearless people afraid of?
Source: Front Psychiatry. 2025 Jun 3;16:1574813. doi: 10.3389/fpsyt.2025.1574813 (PMC12171828; doi:10.3389/fpsyt.2025.1574813)
Supplement: Supplementary file 1 [file Table1.docx]

**Supplementary material 1**

Due to the large number of comparisons and to make the description of the results easier to follow we decided to move the results of groupwise comparisons and descriptive statistics into this supplementary material. The results are presented in tables. First, in Table 1, we present the results of the Games-Howell post-hoc tests. The results are only presented for the main effect that remained significant after the Benjamini-Hochberg false discovery rate correction. Then, in Table 2, we present the central tendencies of each group for all fears.

**Table 1.** The results of the Games-Howell post-hoc tests comparing the three psychopathy groups (Low psychopathy, High primary and High secondary) on all fears on the Fear Survey Schedule.

| **Fear** | **Statistical result** | | |
| --- | --- | --- | --- |
| **Being in a strange place** |  | High primary | High secondary |
| Low psychopathy | Mean difference | 0.0497 | -0.433 |
|  | t-value | 0.403 | -3.72 |
|  | df | 197 | 264 |
|  | p-value | 0.914 | < .001 |
| High primary | Mean difference | — | -0.482 |
|  | t-value | — | -3.66 |
|  | df | — | 216 |
|  | p-value | — | < .001 |
|  |  |  |  |
| **Speaking in public** |  | High primary | High secondary |
| Low psychopathy | Mean difference | 0.327 | -0.400 |
|  | t-value | 44983 | -2.80 |
|  | df | 223 | 271 |
|  | p-value | 0.064 | 0.015 |
| High primary | Mean difference | — | -0.727 |
|  | t-value | — | -4.95 |
|  | df | — | 223 |
|  | p-value | — | < .001 |
|  |  |  |  |
| **Automobiles** |  | High primary | High secondary |
| Low psychopathy | Mean difference | 0.0128 | -0.492 |
|  | t-value | 0.136 | -4.51 |
|  | df | 199 | 225 |
|  | p-value | 0.990 | < .001 |
| High primary | Mean difference | — | -0.505 |
|  | t-value | — | -4.26 |
|  | df | — | 230 |
|  | p-value | — | < .001 |
|  |  |  |  |
| **Being teased** |  | High primary | High secondary |
| Low psychopathy | Mean difference | -0.202 | -0.568 |
|  | t-value | -1.60 | -4.85 |
|  | df | 181 | 253 |
|  | p-value | 0.248 | < .001 |
| High primary | Mean difference | — | -0.365 |
|  | t-value | — | -2.61 |
|  | df | — | 214 |
|  | p-value | — | 0.026 |
|  |  |  |  |
| **Failure** |  | High primary | High secondary |
| Low psychopathy | Mean difference | 0.310 | -0.434 |
|  | t-value | 1.87 | -3.04 |
|  | df | 184 | 268 |
|  | p-value | 0.151 | 0.007 |
| High primary | Mean difference | — | -0.744 |
|  | t-value | — | -4.31 |
|  | df | — | 198 |
|  | p-value | — | < .001 |
|  |  |  |  |
| **Strangers** |  | High primary | High secondary |
| Low psychopathy | Mean difference | 0.00881 | -0.390 |
|  | t-value | 0.0810 | -3.49 |
|  | df | 208 | 257 |
|  | p-value | 0.996 | 0.002 |
| High primary | Mean difference | — | -0.399 |
|  | t-value | — | -3.30 |
|  | df | — | 228 |
|  | p-value | — | 0.003 |
|  |  |  |  |
| **Journey by train** |  | High primary | High secondary |
| Low psychopathy | Mean difference | -0.0734 | -0.1719 |
|  | t-value | -1.52 | -3.58 |
|  | df | 130 | 180 |
|  | p-value | 0.286 | 0.001 |
| High primary | Mean difference | — | -0.0985 |
|  | t-value | — | -1.57 |
|  | df | — | 223 |
|  | p-value | — | 0.261 |
|  |  |  |  |
| **Sudden noises** |  | High primary | High secondary |
| Low psychopathy | Mean difference | 0.0463 | -0.568 |
|  | t-value | 0.375 | -4.41 |
|  | df | 197 | 245 |
|  | p-value | 0.926 | < .001 |
| High primary | Mean difference | — | -0.614 |
|  | t-value | — | -4.29 |
|  | df | — | 229 |
|  | p-value | — | < .001 |
|  |  |  |  |
| **Crowds** |  | High primary | High secondary |
| Low psychopathy | Mean difference | 0.159 | -0.379 |
|  | t-value | 44946 | -2.78 |
|  | df | 229 | 271 |
|  | p-value | 0.457 | 0.016 |
| High primary | Mean difference | — | -0.538 |
|  | t-value | — | -3.97 |
|  | df | — | 228 |
|  | p-value | — | < .001 |
|  |  |  |  |
| **Being watched working** |  | High primary | High secondary |
| Low psychopathy | Mean difference | -0.153 | -0.613 |
|  | t-value | -1.22 | -4.73 |
|  | df | 192 | 243 |
|  | p-value | 0.445 | < .001 |
| High primary | Mean difference | — | -0.460 |
|  | t-value | — | -3.14 |
|  | df | — | 228 |
|  | p-value | — | 0.005 |
|  |  |  |  |
| **Being criticized** |  | High primary | High secondary |
| Low psychopathy | Mean difference | 0.0324 | -0.658 |
|  | t-value | 0.224 | -4.40 |
|  | df | 205 | 254 |
|  | p-value | 0.973 | < .001 |
| High primary | Mean difference | — | -0.691 |
|  | t-value | — | -4.24 |
|  | df | — | 228 |
|  | p-value | — | < .001 |
|  |  |  |  |
| **Angry people** |  | High primary | High secondary |
| Low psychopathy | Mean difference | -0.0430 | -0.454 |
|  | t-value | -0.345 | -3.76 |
|  | df | 198 | 259 |
|  | p-value | 0.936 | < .001 |
| High primary | Mean difference | — | -0.411 |
|  | t-value | — | -3.02 |
|  | df | — | 221 |
|  | p-value | — | 0.008 |
|  |  |  |  |
| **Feeling rejected by others** |  | High primary | High secondary |
| Low psychopathy | Mean difference | 0.128 | -0.647 |
|  | t-value | 0.844 | -4.38 |
|  | df | 203 | 263 |
|  | p-value | 0.676 | < .001 |
| High primary | Mean difference | — | -0.774 |
|  | t-value | — | -4.75 |
|  | df | — | 220 |
|  | p-value | — | < .001 |
|  |  |  |  |
| **Feeling disapproved of** |  | High primary | High secondary |
| Low psychopathy | Mean difference | -0.0908 | -0.738 |
|  | t-value | -0.600 | -5.04 |
|  | df | 188 | 251 |
|  | p-value | 0.820 | < .001 |
| High primary | Mean difference | — | -0.647 |
|  | t-value | — | -3.82 |
|  | df | — | 221 |
|  | p-value | — | < .001 |
|  |  |  |  |
| **Being ignored** |  | High primary | High secondary |
| Low psychopathy | Mean difference | 0.0877 | -0.525 |
|  | t-value | 0.612 | -3.75 |
|  | df | 200 | 260 |
|  | p-value | 0.814 | < .001 |
| High primary | Mean difference | — | -0.613 |
|  | t-value | — | -3.91 |
|  | df | — | 222 |
|  | p-value | — | < .001 |
|  |  |  |  |
| **Nude men** |  | High primary | High secondary |
| Low psychopathy | Mean difference | -0.253 | -0.394 |
|  | t-value | -2.57 | -3.91 |
|  | df | 164 | 215 |
|  | p-value | 0.030 | < .001 |
| High primary | Mean difference | — | -0.141 |
|  | t-value | — | -1.17 |
|  | df | — | 226 |
|  | p-value | — | 0.475 |
|  |  |  |  |
| **Making mistakes** |  | High primary | High secondary |
| Low psychopathy | Mean difference | 0.108 | -0.475 |
|  | t-value | 0.682 | -3.31 |
|  | df | 197 | 269 |
|  | p-value | 0.774 | 0.003 |
| High primary | Mean difference | — | -0.583 |
|  | t-value | — | -3.54 |
|  | df | — | 208 |
|  | p-value | — | 0.001 |
|  |  |  |  |
| **Looking ridiculous** |  | High primary | High secondary |
| Low psychopathy | Mean difference | 0.259 | -0.510 |
|  | t-value | 1.59 | -3.33 |
|  | df | 210 | 271 |
|  | p-value | 0.252 | 0.003 |
| High primary | Mean difference | — | -0.769 |
|  | t-value | — | -4.61 |
|  | df | — | 214 |
|  | p-value | — | < .001 |
|  | | | |

**Table 2.** Central tendencies (mean scores, Standard Deviations and standard Errors) of the three psychopathy groups (Low psychopathy, High primary and High secondary) on all fears on the Fear Survey Schedule.

| **Fear** | **Group** | **N** | **Mean** | **SD** | **SE** |
| --- | --- | --- | --- | --- | --- |
| Noise of vacuum cleaners | Low psychopathy | 139 | 0.0432 | 0.378 | 0.0321 |
|  | High primary | 98 | 0.1122 | 0.428 | 0.0432 |
|  | High secondary | 135 | 0.1259 | 0.495 | 0.0426 |
| Being alone | Low psychopathy | 139 | 0.9496 | 0.919 | 0.0780 |
|  | High primary | 98 | 0.8061 | 0.938 | 0.0947 |
|  | High secondary | 135 | 1.2667 | 1.173 | 0.1010 |
| Being a stranger | Low psychopathy | 139 | 1.1007 | 0.887 | 0.0752 |
|  | High primary | 98 | 1.0510 | 0.967 | 0.0977 |
|  | High secondary | 135 | 1.5333 | 1.028 | 0.0885 |
| Loud voices | Low psychopathy | 139 | 0.5899 | 0.946 | 0.0803 |
|  | High primary | 98 | 0.6429 | 0.876 | 0.0885 |
|  | High secondary | 135 | 1.0148 | 1.222 | 0.1051 |
| Speaking in public | Low psychopathy | 139 | 1.5108 | 1.176 | 0.0997 |
|  | High primary | 98 | 1.1837 | 1.039 | 0.1049 |
|  | High secondary | 135 | 1.9111 | 1.194 | 0.1027 |
| Crossing streets | Low psychopathy | 139 | 0.4245 | 0.625 | 0.0530 |
|  | High primary | 98 | 0.4490 | 0.705 | 0.0712 |
|  | High secondary | 135 | 0.7111 | 0.929 | 0.0800 |
| Automobiles | Low psychopathy | 139 | 0.3597 | 0.681 | 0.0578 |
|  | High primary | 98 | 0.3469 | 0.734 | 0.0741 |
|  | High secondary | 135 | 0.8519 | 1.076 | 0.0926 |
| Being teased | Low psychopathy | 139 | 0.6547 | 0.840 | 0.0713 |
|  | High primary | 98 | 0.8571 | 1.035 | 0.1046 |
|  | High secondary | 135 | 1.2222 | 1.077 | 0.0927 |
| Dentists | Low psychopathy | 139 | 0.5827 | 0.955 | 0.0810 |
|  | High primary | 98 | 0.6939 | 1.009 | 0.1020 |
|  | High secondary | 135 | 0.9556 | 1.227 | 0.1056 |
| Failure | Low psychopathy | 139 | 2.1367 | 1.124 | 0.0954 |
|  | High primary | 98 | 1.8265 | 1.347 | 0.1361 |
|  | High secondary | 135 | 2.5704 | 1.231 | 0.1060 |
| Entering a room where other people are already seated | Low psychopathy | 139 | 0.7266 | 0.891 | 0.0756 |
|  | High primary | 98 | 0.8163 | 1.039 | 0.1049 |
|  | High secondary | 135 | 1.0074 | 1.175 | 0.1011 |
| People with deformities | Low psychopathy | 139 | 0.3381 | 0.676 | 0.0574 |
|  | High primary | 98 | 0.5102 | 0.815 | 0.0824 |
|  | High secondary | 135 | 0.4519 | 0.740 | 0.0637 |
| Worms | Low psychopathy | 139 | 1.1942 | 1.203 | 0.1020 |
|  | High primary | 98 | 1.0408 | 1.192 | 0.1204 |
|  | High secondary | 135 | 1.4741 | 1.239 | 0.1066 |
| Imaginary creatures | Low psychopathy | 139 | 0.3669 | 0.714 | 0.0605 |
|  | High primary | 98 | 0.5918 | 1.014 | 0.1024 |
|  | High secondary | 135 | 0.7111 | 1.099 | 0.0946 |
| Receiving injections | Low psychopathy | 139 | 0.5755 | 0.940 | 0.0797 |
|  | High primary | 98 | 0.6531 | 1.026 | 0.1037 |
|  | High secondary | 135 | 0.9481 | 1.289 | 0.1109 |
| Strangers | Low psychopathy | 139 | 0.6619 | 0.821 | 0.0697 |
|  | High primary | 98 | 0.6531 | 0.826 | 0.0835 |
|  | High secondary | 135 | 1.0519 | 1.017 | 0.0875 |
| Bats | Low psychopathy | 139 | 0.8561 | 1.033 | 0.0876 |
|  | High primary | 98 | 0.8469 | 1.039 | 0.1050 |
|  | High secondary | 135 | 1.2519 | 1.286 | 0.1106 |
| Journey by train | Low psychopathy | 139 | 0.0504 | 0.219 | 0.0186 |
|  | High primary | 97 | 0.1237 | 0.439 | 0.0446 |
|  | High secondary | 135 | 0.2222 | 0.513 | 0.0442 |
| Journey by bus | Low psychopathy | 139 | 0.1007 | 0.386 | 0.0328 |
|  | High primary | 98 | 0.1531 | 0.484 | 0.0489 |
|  | High secondary | 135 | 0.2963 | 0.647 | 0.0557 |
| Journey by car | Low psychopathy | 139 | 0.1367 | 0.385 | 0.0326 |
|  | High primary | 98 | 0.1837 | 0.484 | 0.0489 |
|  | High secondary | 135 | 0.3778 | 0.762 | 0.0656 |
| Feeling angry | Low psychopathy | 139 | 0.8993 | 0.943 | 0.0799 |
|  | High primary | 98 | 0.9490 | 1.106 | 0.1118 |
|  | High secondary | 135 | 1.1481 | 1.237 | 0.1065 |
| People in authority | Low psychopathy | 139 | 0.4317 | 0.671 | 0.0569 |
|  | High primary | 98 | 0.6224 | 0.819 | 0.0827 |
|  | High secondary | 135 | 0.6444 | 0.885 | 0.0762 |
| Flying insects | Low psychopathy | 139 | 0.8993 | 1.045 | 0.0886 |
|  | High primary | 98 | 1.0204 | 1.065 | 0.1076 |
|  | High secondary | 134 | 1.3657 | 1.192 | 0.1030 |
| Seeing other people being injected | Low psychopathy | 139 | 0.5396 | 0.973 | 0.0825 |
|  | High primary | 98 | 0.6429 | 1.028 | 0.1038 |
|  | High secondary | 135 | 0.8741 | 1.248 | 0.1075 |
| Sudden noises | Low psychopathy | 139 | 0.9137 | 0.889 | 0.0754 |
|  | High primary | 98 | 0.8673 | 0.970 | 0.0980 |
|  | High secondary | 135 | 1.4815 | 1.215 | 0.1045 |
| Dull weather | Low psychopathy | 139 | 0.6906 | 0.916 | 0.0777 |
|  | High primary | 98 | 0.6531 | 0.932 | 0.0941 |
|  | High secondary | 135 | 0.8370 | 1.173 | 0.1010 |
| Crowds | Low psychopathy | 139 | 0.7914 | 1.113 | 0.0944 |
|  | High primary | 98 | 0.6327 | 0.924 | 0.0933 |
|  | High secondary | 135 | 1.1704 | 1.143 | 0.0984 |
| Large open spaces | Low psychopathy | 139 | 0.1223 | 0.370 | 0.0314 |
|  | High primary | 98 | 0.1224 | 0.413 | 0.0417 |
|  | High secondary | 135 | 0.1481 | 0.415 | 0.0357 |
| Cats | Low psychopathy | 139 | 0.2374 | 0.609 | 0.0516 |
|  | High primary | 98 | 0.3367 | 0.608 | 0.0614 |
|  | High secondary | 135 | 0.3778 | 0.809 | 0.0696 |
| One person bullying another | Low psychopathy | 139 | 0.8417 | 0.870 | 0.0738 |
|  | High primary | 98 | 0.8776 | 1.038 | 0.1049 |
|  | High secondary | 135 | 1.0815 | 1.037 | 0.0893 |
| Tough looking people | Low psychopathy | 139 | 0.7626 | 0.873 | 0.0740 |
|  | High primary | 98 | 0.7347 | 0.926 | 0.0935 |
|  | High secondary | 135 | 0.9704 | 0.922 | 0.0793 |
| Birds | Low psychopathy | 139 | 1.4676 | 1.281 | 0.1087 |
|  | High primary | 98 | 1.1020 | 1.248 | 0.1260 |
|  | High secondary | 135 | 1.4296 | 1.273 | 0.1095 |
| Deepwater | Low psychopathy | 139 | 1.7554 | 1.307 | 0.1108 |
|  | High primary | 98 | 1.8163 | 1.438 | 0.1453 |
|  | High secondary | 135 | 2.0074 | 1.453 | 0.1251 |
| Being watched working | Low psychopathy | 139 | 0.6835 | 0.885 | 0.0751 |
|  | High primary | 98 | 0.8367 | 1.002 | 0.1012 |
|  | High secondary | 135 | 1.2963 | 1.228 | 0.1057 |
| Dead animals | Low psychopathy | 139 | 0.9281 | 1.114 | 0.0945 |
|  | High primary | 98 | 1.0510 | 1.279 | 0.1292 |
|  | High secondary | 135 | 1.1556 | 1.251 | 0.1077 |
| Weapons | Low psychopathy | 139 | 1.8345 | 1.472 | 0.1249 |
|  | High primary | 98 | 1.5612 | 1.393 | 0.1407 |
|  | High secondary | 135 | 1.9185 | 1.425 | 0.1226 |
| Dirt | Low psychopathy | 139 | 0.8417 | 1.030 | 0.0874 |
|  | High primary | 98 | 0.6735 | 0.972 | 0.0981 |
|  | High secondary | 135 | 0.7852 | 1.047 | 0.0901 |
| Crawling insects | Low psychopathy | 139 | 1.5468 | 1.303 | 0.1106 |
|  | High primary | 98 | 1.4898 | 1.326 | 0.1339 |
|  | High secondary | 135 | 1.8444 | 1.371 | 0.1180 |
| Sight of fighting | Low psychopathy | 139 | 1.2014 | 1.292 | 0.1096 |
|  | High primary | 98 | 1.1224 | 1.195 | 0.1207 |
|  | High secondary | 135 | 1.5926 | 1.368 | 0.1177 |
| Ugly people | Low psychopathy | 139 | 0.1727 | 0.510 | 0.0432 |
|  | High primary | 98 | 0.2143 | 0.646 | 0.0653 |
|  | High secondary | 135 | 0.2889 | 0.742 | 0.0638 |
| Fire | Low psychopathy | 139 | 1.5683 | 1.414 | 0.1200 |
|  | High primary | 98 | 1.4694 | 1.245 | 0.1258 |
|  | High secondary | 135 | 1.7481 | 1.428 | 0.1229 |
| Sick people | Low psychopathy | 139 | 0.7986 | 1.037 | 0.0879 |
|  | High primary | 98 | 0.9082 | 1.185 | 0.1197 |
|  | High secondary | 135 | 1.0444 | 1.190 | 0.1024 |
| Dogs | Low psychopathy | 139 | 0.5252 | 0.846 | 0.0717 |
|  | High primary | 98 | 0.4490 | 0.863 | 0.0872 |
|  | High secondary | 135 | 0.7333 | 1.154 | 0.0993 |
| Being criticized | Low psychopathy | 139 | 0.9712 | 1.076 | 0.0913 |
|  | High primary | 98 | 0.9388 | 1.111 | 0.1122 |
|  | High secondary | 135 | 1.6296 | 1.375 | 0.1184 |
| Strange shapes | Low psychopathy | 139 | 0.3813 | 0.765 | 0.0649 |
|  | High primary | 98 | 0.4490 | 0.801 | 0.0809 |
|  | High secondary | 135 | 0.7185 | 0.944 | 0.0812 |
| Being in an elevator | Low psychopathy | 139 | 0.5899 | 0.962 | 0.0816 |
|  | High primary | 98 | 0.5816 | 0.907 | 0.0916 |
|  | High secondary | 135 | 0.7704 | 1.126 | 0.0969 |
| Witnessing surgical operations | Low psychopathy | 139 | 1.1367 | 1.358 | 0.1152 |
|  | High primary | 98 | 1.1327 | 1.265 | 0.1278 |
|  | High secondary | 135 | 1.4444 | 1.444 | 0.1243 |
| Angry people | Low psychopathy | 139 | 0.8345 | 0.898 | 0.0761 |
|  | High primary | 98 | 0.8776 | 0.977 | 0.0987 |
|  | High secondary | 135 | 1.2889 | 1.092 | 0.0940 |
| Mice | Low psychopathy | 139 | 1.1439 | 1.254 | 0.1064 |
|  | High primary | 98 | 1.3061 | 1.342 | 0.1356 |
|  | High secondary | 135 | 1.2444 | 1.212 | 0.1043 |
| Human blood | Low psychopathy | 139 | 0.7770 | 1.117 | 0.0947 |
|  | High primary | 98 | 0.7551 | 1.016 | 0.1026 |
|  | High secondary | 135 | 0.8370 | 1.179 | 0.1015 |
| Animal blood | Low psychopathy | 139 | 0.7194 | 1.036 | 0.0879 |
|  | High primary | 98 | 0.7143 | 0.942 | 0.0951 |
|  | High secondary | 135 | 0.8741 | 1.181 | 0.1016 |
| Parting from friends | Low psychopathy | 139 | 1.4029 | 1.178 | 0.0999 |
|  | High primary | 98 | 1.3469 | 1.228 | 0.1240 |
|  | High secondary | 135 | 1.6667 | 1.240 | 0.1067 |
| Enclosed places | Low psychopathy | 139 | 0.7482 | 1.050 | 0.0891 |
|  | High primary | 98 | 0.9286 | 1.186 | 0.1198 |
|  | High secondary | 135 | 1.0519 | 1.318 | 0.1134 |
| Feeling rejected by others | Low psychopathy | 139 | 1.3237 | 1.124 | 0.0954 |
|  | High primary | 97 | 1.1959 | 1.160 | 0.1178 |
|  | High secondary | 135 | 1.9704 | 1.310 | 0.1127 |
| Airplanes | Low psychopathy | 139 | 0.5468 | 0.950 | 0.0805 |
|  | High primary | 98 | 0.8571 | 1.131 | 0.1142 |
|  | High secondary | 135 | 0.9778 | 1.261 | 0.1085 |
| Medical odors | Low psychopathy | 139 | 0.3957 | 0.777 | 0.0659 |
|  | High primary | 98 | 0.6224 | 0.936 | 0.0946 |
|  | High secondary | 135 | 0.6148 | 1.072 | 0.0923 |
| Feeling disapproved of | Low psychopathy | 139 | 1.1439 | 1.040 | 0.0882 |
|  | High primary | 98 | 1.2347 | 1.217 | 0.1229 |
|  | High secondary | 135 | 1.8815 | 1.355 | 0.1166 |
| Harmless snakes | Low psychopathy | 139 | 1.4245 | 1.464 | 0.1242 |
|  | High primary | 98 | 1.5408 | 1.528 | 0.1543 |
|  | High secondary | 135 | 1.8519 | 1.504 | 0.1294 |
| Cemeteries | Low psychopathy | 139 | 0.7122 | 0.995 | 0.0844 |
|  | High primary | 98 | 0.9490 | 1.213 | 0.1225 |
|  | High secondary | 135 | 0.9556 | 1.119 | 0.0963 |
| Being ignored | Low psychopathy | 139 | 0.9856 | 1.042 | 0.0884 |
|  | High primary | 98 | 0.8980 | 1.117 | 0.1128 |
|  | High secondary | 135 | 1.5111 | 1.263 | 0.1087 |
| Darkness | Low psychopathy | 139 | 0.9281 | 1.121 | 0.0950 |
|  | High primary | 98 | 0.9082 | 1.036 | 0.1047 |
|  | High secondary | 135 | 1.1926 | 1.231 | 0.1059 |
| Premature heartbeat/missing a beat | Low psychopathy | 139 | 0.9640 | 1.113 | 0.0944 |
|  | High primary | 98 | 1.1939 | 1.207 | 0.1219 |
|  | High secondary | 135 | 1.1704 | 1.219 | 0.1049 |
| Nude men | Low psychopathy | 139 | 0.1655 | 0.597 | 0.0506 |
|  | High primary | 98 | 0.4184 | 0.836 | 0.0845 |
|  | High secondary | 134 | 0.5597 | 1.008 | 0.0870 |
| Nude women | Low psychopathy | 139 | 0.1151 | 0.435 | 0.0369 |
|  | High primary | 98 | 0.3061 | 0.779 | 0.0787 |
|  | High secondary | 135 | 0.2444 | 0.592 | 0.0509 |
| Lightning | Low psychopathy | 139 | 0.7410 | 1.092 | 0.0927 |
|  | High primary | 98 | 0.8061 | 1.146 | 0.1157 |
|  | High secondary | 135 | 0.8444 | 1.196 | 0.1030 |
| Doctors | Low psychopathy | 139 | 0.2086 | 0.558 | 0.0473 |
|  | High primary | 98 | 0.3163 | 0.619 | 0.0626 |
|  | High secondary | 135 | 0.4519 | 0.944 | 0.0813 |
| Making mistakes | Low psychopathy | 139 | 1.5468 | 1.137 | 0.0965 |
|  | High primary | 98 | 1.4388 | 1.244 | 0.1257 |
|  | High secondary | 135 | 2.0222 | 1.237 | 0.1064 |
| Looking ridiculous | Low psychopathy | 139 | 1.4532 | 1.241 | 0.1052 |
|  | High primary | 98 | 1.1939 | 1.232 | 0.1245 |
|  | High secondary | 135 | 1.9630 | 1.289 | 0.1110 |
| Supernatural | Low psychopathy | 139 | 1.0216 | 1.201 | 0.1018 |
|  | High primary | 98 | 1.0000 | 1.193 | 0.1205 |
|  | High secondary | 135 | 1.3852 | 1.451 | 0.1248 |
| Bacteria/Virus | Low psychopathy | 139 | 1.5036 | 1.259 | 0.1068 |
|  | High primary | 98 | 1.3265 | 1.283 | 0.1296 |
|  | High secondary | 135 | 1.5926 | 1.248 | 0.1074 |
| Open wounds | Low psychopathy | 139 | 0.9281 | 1.108 | 0.0939 |
|  | High primary | 98 | 0.9694 | 1.020 | 0.1030 |
|  | High secondary | 135 | 1.2222 | 1.124 | 0.0968 |
| Dead people | Low psychopathy | 139 | 1.5971 | 1.402 | 0.1190 |
|  | High primary | 98 | 1.5000 | 1.438 | 0.1452 |
|  | High secondary | 135 | 1.7481 | 1.413 | 0.1216 |
| People who seem insane | Low psychopathy | 139 | 1.4245 | 1.063 | 0.0902 |
|  | High primary | 98 | 1.2959 | 1.168 | 0.1180 |
|  | High secondary | 135 | 1.6000 | 1.121 | 0.0965 |
| Falling | Low psychopathy | 139 | 1.2446 | 1.096 | 0.0929 |
|  | High primary | 98 | 1.2449 | 1.131 | 0.1143 |
|  | High secondary | 135 | 1.4963 | 1.215 | 0.1046 |
| Thunders | Low psychopathy | 139 | 0.7194 | 1.070 | 0.0908 |
|  | High primary | 98 | 0.8265 | 1.103 | 0.1114 |
|  | High secondary | 135 | 0.9037 | 1.233 | 0.1061 |
| Sirens | Low psychopathy | 139 | 0.4029 | 0.768 | 0.0652 |
|  | High primary | 98 | 0.4184 | 0.785 | 0.0793 |
|  | High secondary | 135 | 0.6000 | 0.994 | 0.0856 |
| High places on land | Low psychopathy | 139 | 1.4964 | 1.337 | 0.1134 |
|  | High primary | 98 | 1.5510 | 1.293 | 0.1306 |
|  | High secondary | 135 | 1.7778 | 1.391 | 0.1197 |
|  | | | | | |
|  |  |  |  |  |  |
